# The Science of Bedroom Fires

**Published:** 1888-02-25

**Authors:** 


### THE DOCTOR'S PULPIT.

### THE SCIENCE OF BEDROOM FIRES.

OUR country grandmothers—deft-handed, self-reliant women—had a magnificent contempt for bedroom fires and for those who indulged in them. If a healthy man could not go to bed in the cold and plunge into a pair of fine linen sheets which, except for their dryness, might have been made of very thin ice, he was a poor delicate creature, entirely unfit for daily battle in a rough-and-tumble world like this. Custom and tradition die hard; and there are many descendants of those self-same grandmothers who, although they do not absolutely prohibit bedroom fires, yet have an uneasy suspicion that they are not healthy; that at any rate they do not "harden" the sleeper; and that in fact they are of the nature of a weak-kneed concession to the feebleness and self-indulgence of the present generation.

Our village ancestors had many rare qualities; but they were possibly sometimes wrong in their elementary natural philosophy. Is a wool shirt better than an unprotected skin? Is a warm overcoat *plus* the wool shirt better than the shirt alone when the thermometer shows twenty degrees of frost? Is a bare barn in a snowstorm more comfortable than the open moor? And is the best room in the village inn, with the landlady's fattest fowl done to a turn, and toast and tea with fresh cream, better than the bare barn? All the arguments which may be advanced in favour of the wool shirt and the overcoat, the barn and the inn's cosy parlour, may be advanced with almost equal propriety and force in favour of bed-room fires in the winter.

What does natural science say on the point? It says that when a bedroom never has a fire in it the air becomes exceedingly cold, and what is more, that it becomes exceedingly stagnant. Cold stagnant air is not only difficult to breathe but injurious when breathed. A man enters a bedroom which has no fire in it at eleven o'clock on a keen February night. The thermometer marks ten or twelve degrees of frost. Why does he begin to sneeze violently before he has been two minutes in the room? Because the frosty air passing over the mucous membrane of his nostrils irritates the fine nerve-endings, which it ought not to do, and they instantly make the most vigorous protest they can in the shape of a sneezing fit. He begins to shiver before he is half undressed, and with chattering teeth jumps into an icy and uncivilized bed. In less than a minute he is chilled to the very marrow. The enfolding, death-like sheets, the raw, foggy air rushing into the very centre of the circulation in the lungs, co-operate to produce a condition of benumbed sensibility which no man of cultivated taste will ever voluntarily put himself into. Of course, if a man have the activity of a wild lion, the natural fur of a polar bear, and the digestion of a rhinoceros, he may sleep in any bed or bedroom he pleases, even at the North Pole. But ordinary mortals are not so blessed, and it behoves them to exercise a reasonable degree of sense on these questions.

Science says further that the air in unwarmed bed-rooms is not *fresh;* that it can never be half so fresh and pure as when a good fire is kept. This is very easy of explanation, When a fire is burning in the grate the fire itself uses up a considerable quantity of atmospheric air in the act of burning. Not only so, but the chimney becomes a hot shaft, carrying up, as draught, a large quantity of air. Where does the air come from which is used in burning, or passes up the chimney? It can only come from inside the room. As rapidly as the air passes up the chimney, so rapidly is a vacant space made in the room, and so rapidly also does the air outside the house rush in by every chink and cranny and opening, and even through the walls, to fill up the vacant space. For it must be remembered that the atmosphere is always pressing on every house and window, and at every possible opening, with a pressure of fifteen pounds to the square inch. The fireless bed-room may be compared to a castle in the hands of an enemy, the cold *stagnant* air being the enemy. At the windows and other openings peeps in a friend in the shape of cold *fresh* air. But so long as the air inside is of the same temperature as that outside its pressure is also the same, and so the inside air holds the castle. Now let the maid enter and light a brisk fire, and immediately some of the air of the room is used up for combustion, more passes up the chimney in the current, and all the rest is well warmed, and its power of resistance to the outer air is diminished in exact proportion to its increased warmth. So that a fire rapidly clears the room of an enemy in the shape of cold, stagnant air, and brings in a friend in the shape of fresh air, which is soon warmed. That is the teaching of simple, demonstrable science.

Some people are not convinced by science; they stand by experience. To experience, then, let the appeal be made. Only, we may premise that when what is called science appears to contradict experience, the so-called science *may* be wrong; but when what is called experience contradicts demonstrable science, the experience *must* be wrong. In the third possible case, where science and experience are absolutely agreed, the objector has nothing to do but to admit himself defeated. If he is not convinced as well as defeated, he certainly is no philosopher.

What then does experience say on this very practical question? It says that a cold bed in a cold room produces exceedingly uncomfortable sensations at midnight in the end of February, as any reader may prove for himself when he goes to bed to-night. It says that cold stagnant air is very difficult to breathe; and it proves this constantly when an asthmatical man brings on an acute attack by going to a cold bed, and cures his acute attack by having a good fire lighted and drinking a large cup of very hot coffee. It says that when a man is suffering from bronchitis or pneumonia, one of the first things done for his relief is to light a fire in his room and to charge the air with hot steam as rapidly as possible. It says that a patient with a chronic cough may get no sleep all night if the maid neglects to put a good fire in his room two hours before he goes to bed; but that if she makes the room cosy, and the bed warm with nice hot bottles, he will very likely go off to sleep at once and wake no more until breakfast-time. It says that a poor old lady with a weak heart will wheeze and pant and be unable even to lie down in a cold and miserable sleeping apartment, whilst the same old lady will be tranquil and happy in a temperature of sixty-eight degrees and with a glass of hot toddy for a "nightcap." Experience—genuine experience—is a fine and wise teacher, and never by any chance disagrees with true science. Pseudo-science, scientific chatter, scientific pomposity, and all that kind of thing, will often affect to despise and contemn experience, and when it does so, experience may go on her way and laugh at it; or, if she pleases, she may prick it with a pin and let the air out of the windbag. But true science and true experience are twin sisters, and were never known to quarrel since the world began. He who is wise will make himself the disciple and friend of both.

